# Noise-Resilient Depth Estimation for Light Field Images Using Focal Stack and FFT Analysis

**DOI:** 10.3390/s22051993

**Published:** 2022-03-03

**Authors:** Rishabh Sharma, Stuart Perry, Eva Cheng

**Affiliations:** School of Electrical and Data Engineering, University of Technology Sydney, Ultimo, NSW 2007, Australia; stuart.perry@uts.edu.au (S.P.); eva.cheng@uts.edu.au (E.C.)

**Keywords:** depth map, focus map, light field, focal stack

## Abstract

Depth estimation for light field images is essential for applications such as light field image compression, reconstructing perspective views and 3D reconstruction. Previous depth map estimation approaches do not capture sharp transitions around object boundaries due to occlusions, making many of the current approaches unreliable at depth discontinuities. This is especially the case for light field images because the pixels do not exhibit photo-consistency in the presence of occlusions. In this paper, we propose an algorithm to estimate the depth map for light field images using depth from defocus. Our approach uses a small patch size of pixels in each focal stack image for comparing defocus cues, allowing the algorithm to generate sharper depth boundaries. Then, in contrast to existing approaches that use defocus cues for depth estimation, we use frequency domain analysis image similarity checking to generate the depth map. Processing in the frequency domain reduces the individual pixel errors that occur while directly comparing RGB images, making the algorithm more resilient to noise. The algorithm has been evaluated on both a synthetic image dataset and real-world images in the JPEG dataset. Experimental results demonstrate that our proposed algorithm outperforms state-of-the-art depth estimation techniques for light field images, particularly in case of noisy images.

## 1. Introduction

Depth estimation for planar images is essential for applications such as changing depth of focus, simulating subsurface scattering and shadow mapping. Depth estimation is also crucial in computer vision applications such as robot vision, self-driving cars, surveillance and human–computer interactions. Depth estimation algorithms also play an important role in semantic segmentation algorithms [1,2]. To estimate depth maps, a wide range of stereo matching techniques have been proposed and implemented, and Scharstein and Szeliski present an in-depth analysis of these techniques [3]. In recent years, the introduction of light field images has made it possible to generate images at different focal lengths, extended depth of field and estimate the scene depth from a single image capture. Depth maps from light field images are essential for light field image compression techniques, reconstructing views from a sparse set of perspective views, increasing the number of perspective views and 3D reconstruction.

Compared to the techniques using a single image or stereo image pair for depth estimation, light field images enable researchers to explore techniques such as depth from correspondence and defocus from a single image. This is due to light field images capturing not only the pixel intensity but also the direction of the incident light with a single capture; however, the challenges in depth map estimation from planar images still apply, and are compounded by the challenges of estimating depth from light field images, including the presence of occlusion, textureless regions and overexposed regions in images.

**Figure 1:**
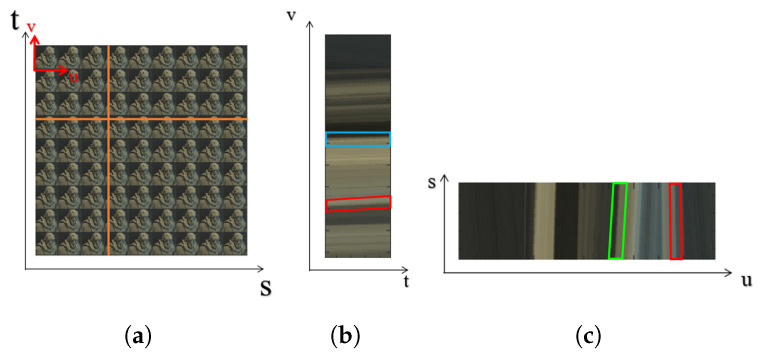
(**a**) The sub-aperture image view; (**b**) the EPI for the vertical line represented in (**a**); (**c**) the EPI for the horizontal line represented in (**a**).

Existing light field depth estimation techniques include stereo-based matching, using epipolar images, depth from defocus and more recent techniques that use neural networks. Stereo matching for depth estimation suffers from ambiguity due to partial occlusions [4]. Since the stereo pair are images, the information lost by the occlusions cannot be recovered but can only be approximated using a smoothness term to fill the gaps. In contrast, epipolar images are formed by stacking together the sub-aperture images in both the horizontal and vertical direction as shown in Figure 1b,c, respectively. A slice through this 4D block reveals the depth of the pixels, as the slope of the line reflects the depth information as shown by the red, green and blue parallelograms in Figure 1. The pixels in the central view that do not move with changing sub-aperture views have a zero slope and are seen as a straight line as shown by the blue parallelograms in Figure 1b. The pixels that are closer to the camera incline to the right as shown by the green parallelograms in Figure 1c, and the pixels that are further away from the camera incline to the left as shown by the red parallelograms in Figure 1. However, basic line fitting techniques to estimate depth would not give robust results and the reconstructions are generally noisy [5]. Schechner and Kiryati [6] have extensively studied depth from defocus and correspondence techniques and have compared the advantages and disadvantages of both cues. Finally, Convolutional Neural Networks (CNN) are a known approach in imaging applications such as object recognition, human–computer interaction, image segmentation and stereo depth estimation [7]. However, the lack of training data in existing light field images datasets makes it hard to train and test the network for light field images [8].

In this paper, we exploit the characteristic of light field images having multiple focal planes that are captured in a single image to generate the disparity or depth map. Our algorithm is designed to use the concept of depth from defocus by a one-to-one comparison between the focal stack image and the central all-in-focus image. We show that our approach is noise resilient in depth estimation, and out-performs the current state-of-the-art benchmark algorithms in the presence of noise. The proposed algorithm is compared to four benchmark techniques from Strecke et al. [9], Wang et al. [10], Zhang et al. [11] and Shin et al. [12] for synthetic images with and without noise. All the four techniques discuss their algorithms’ noise resilience in their work. Strecke et al. [9] suggest mixing the focal stack cost volume with a stereo correspondence cost volume to increase resilience against noise. Wang et al. [10] validate the robustness of their system on noisy image, and report the F-measure values of each algorithm. Zhang et al. [11] suggest reducing the number of bins for depth estimation in the presence of noise, but do not compare their algorithm’s noise performance result to other algorithms. The multi-stream CNN proposed in Shin et al. [12] uses 2 × 2 kernel that reduces the effect of noise.

## 2. Our Contribution

The main contributions of our work are:

1. To reduce the dependence of depth accuracy on RGB values of individual pixels compared in the image patches, we propose a method that uses frequency domain analysis to estimate the depth map for light field images.
2. The key contribution of our approach is noise resilience in depth estimation. Our analysis confirms the hypothesis that comparing focal stack image patches in the frequency domain improves depth map accuracy, especially in the presence of noise. We shown that our algorithm out-performs the current state-of-the-art benchmark algorithms in the presence of noise.

## 3. Background

**Figure 2:**
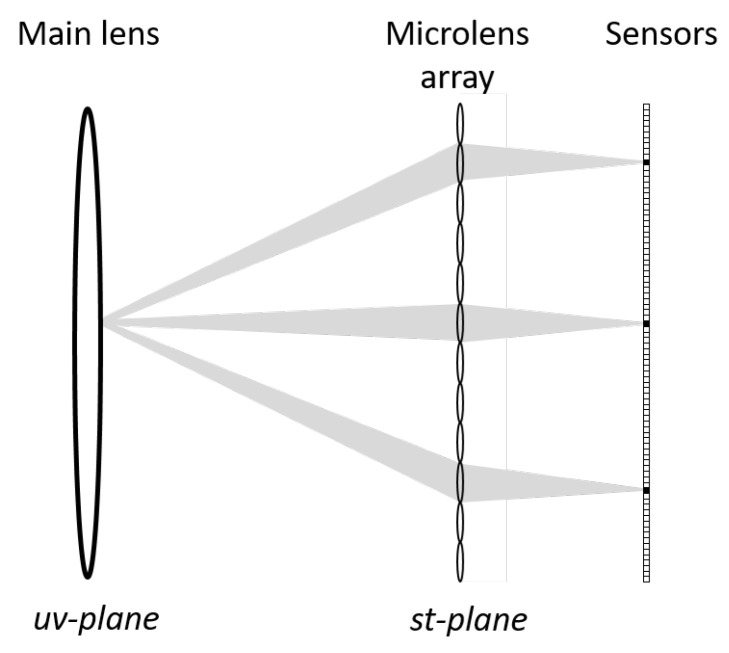
Diagrammatic representation of a lenslet-based plenoptic camera to capture light field images [17].

Conventional photography is only able to capture limited information from the light passing through the camera lens. In general, cameras record the sum of the intensities of light rays striking each point in the image and not the total amount of incident light traveling along different rays that contribute to the image [13]. In contrast, light field imaging technology captures rich visual information by representing the distribution of light in free space [14], which means that a light field image captures the pixel intensity and the direction of the incident light. The additional dimensions of data captured enables the generation of images at different focal lengths and extended depth of field using ray-tracing techniques. This allows for image manipulation in a more flexible way [15]. Capturing a light field can be a challenging task as a light field image captures the pixel intensity and the direction of the incident light. The two techniques that can be used to capture a light field image are: first, using an array of video cameras as described in [16]; then second, using a plenoptic camera as shown in Figure 2. Once the light field image is captured, raytracing can be applied to filter out the light rays that do not contribute to a particular depth. Rearranging the rays then estimates where the light rays would terminate if the camera was focused on the desired depth [17].

**Figure 3:**
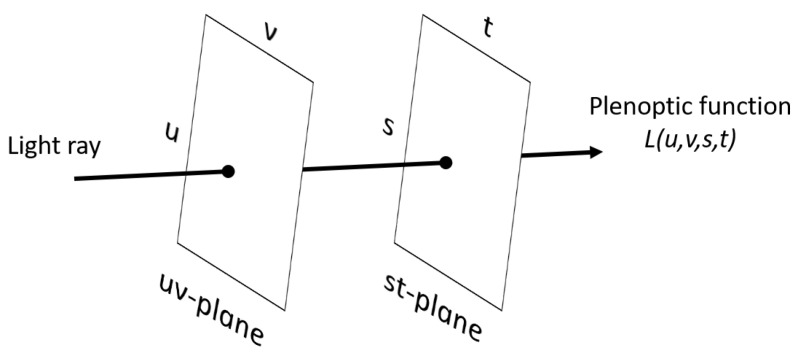
Schematic representation of the plenoptic function [18].

The model that describes the distribution of light is known as a plenoptic function. The plenoptic function describes light as a function of position, angle of incidence, wavelength and time [14]. As shown in Figure 3, the most common way to represent the 4D plenoptic function is to parameterize the light rays as an intersection of the light ray on two planes placed at arbitrary positions [14]. The plenoptic function can thus be defined as *L* (*u*, *v*, *s*, *t*); where (*u*, *v*) and (*s*, *t*) denote the position of intersection of the light ray on the two planes, respectively. The function *L* (*u*, *v*, *s*, *t*) is a two-plane model of light field in which the *st* plane can be considered as a set of cameras that have the focal plane on the *uv* plane [14].

**Figure 4:**
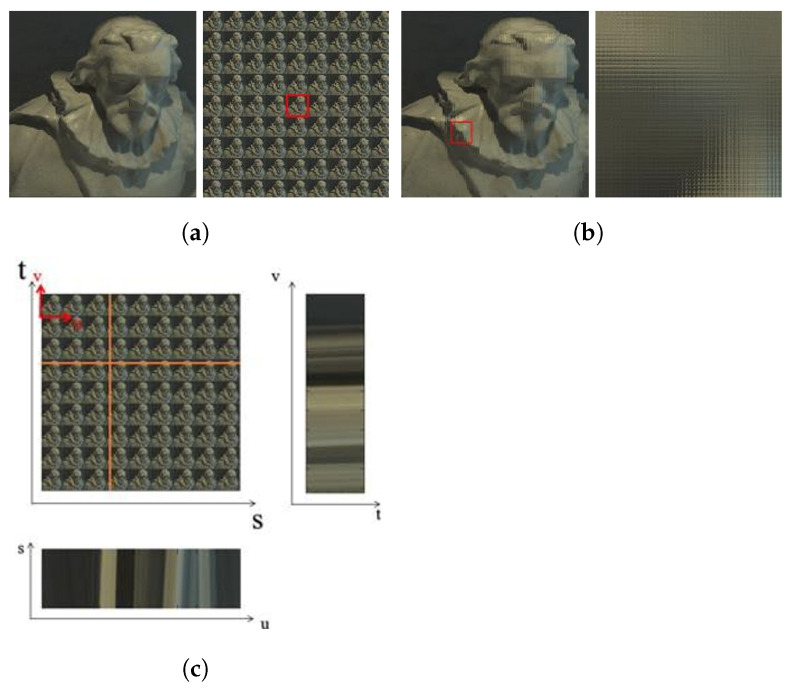
The three representations of the two-plane light field. (**a**) The sub-aperture images or the pinhole view. (**b**) The sub-view for the light field. (**c**) Epipolar-plane image, which is obtained by fixing both spatial and angular co-ordinates [18].

Finally, a light field image can be visualized by representing the image as an Epipolar Plane Image (EPI). The EPI can be obtained by fixing one coordinate in both spatial and angular domains, while plotting pixel intensities as the other coordinates of the spatial and angular domain are varied [11], as shown in Figure 4c.

## 4. Related Work

Current key approaches to light field depth estimation techniques include stereo based matching, using epipolar images, depth from defocus and, more recently, the use of Convolutional Neural Networks (CNNs).

### 4.1. Depth Estimation Using Stereo Matching

Many of the proposed stereo matching algorithms are based on graph cuts and energy minimization techniques by using different constraints. Kolmogorov and Zabih [19] and Woodford et al. [20] combine visibility and smoothness terms for effective optimization Bleyer et al. [21], on the other hand, consider the pixel appearance, global MDL constraint, smoothing, soft segmentation, surface curvature and occlusion. However, the stereo correspondence methods suffer from ambiguities while dealing with noisy and aliased regions, and the narrow baseline for real camera LF images makes it difficult for these algorithms to solve the occlusion problem [11].

### 4.2. Depth Estimation Using Epipolar Plane Images

Depth estimation using Epipolar Plane Images (EPI) is another popular approach. Each slice through the 4D light field representation can be used to analyze the line structure to estimate the depth of the pixel under inspection as shown in Figure 1a,b. Johannsen et al. [22] propose a method that learns the structural information from the central view, and compare them to the 2D 5 × 5 epipolar image patch to estimate the depth map. Wanner and Goldluecke [23] address the problem of reflective surfaces in the image that cause irregularities in the epipolar plane. They propose the use of higher order structure tensors to overcome this problem. Criminisi et al. [24] use the epipolar images to build and minimize the matching cost for each pixel that accounts for the pixel intensity value, gradient pixel value and spatial consistency. Criminisi et al. [24] use canny edge operator, while Wanner and Goldluecke [25] use structure tensor to obtain local disparity estimates by computing the weighted mean of each side of the line in the EPIs and then finding the distance between the two means [11]; however, occlusions and noise could cause the pixels on both sides of the lines to be different. Zhang et al. [11] propose a spinning parallelogram operator that tries to estimate the local direction of a line at a specific point in an EPI, and to avoid problem of the inconsistency of pixel distribution on either sides of the line, they consider each side separately. This assumption makes their depth estimation algorithm more robust to occlusions and noise, then Criminisi et al. [24] and Wanner and Goldluecke [25]’s algorithm. We compare our results with Zhang et al. [11] for noisy images and their EPI-based technique generates outliers that severely effects the accuracy of their depth maps.

### 4.3. Depth Estimation Using Defocus

Depth estimation from defocus has been studied extensively e.g., Schechner and Kiryati [6], who compare the advantages and disadvantages of using defocus or correspondence cues. Research on depth from defocus also extends beyond using LF images, estimating the depth map using a single image [26,27]. Nayar and Nakagawa [28] in their work demonstrate that the defocused imaging system plays the role of a low-pass filter to estimate depth using the measure of focus between image points. Strecke et al. [9] create four separate stacks using only the views right of, left of, above and below the reference view. The assumption is that the baseline is small enough so that if occlusion is present it occurs only in one direction of view point shift. Tao et al. [29] proposed to combine the defocus and correspondence cues using a MRF as the global optimization process. Building on the concept of combining defocus and correspondence cues for depth estimation [29], Wang et al. [10] take into account occlusion to estimate a more accurate depth map. To account for occlusion, an angular patch is generated for the reference pixel to then check for the photo-consistency of that patch. However, similar to other proposed techniques, the algorithm does not perform well in regions of low texture [10].

### 4.4. Depth Estimation Using Convolutional Neural Networks

Convolutional Neural Networks (CNN) have been used extensively in image processing applications such as object recognition, human–computer interaction, image segmentation and stereo depth estimation [7]. Over the past few years, research has been conducted to use CNN for depth estimation in light field images [7,8,30]. The main concern in using CNN for light field images is that the existing light field datasets are insufficient in size to train the network, and datasets do not have accurate ground truth depth included [30]. To address this problem, Heber and Pock [30] generated a synthetic light field dataset using the raytracing software POV-Ray, Feng et al. [7] use the Lytro camera to capture the light field images and then use a 3dMD scanner [31] to capture the ground truth, Shin et al. [12] on the other hand augment the data through scaling, center view change, rotation, transpose and color that are suitable for light field images. In their approach, Heber and Pock [30] extract the information from the vertical and horizontal EPIs to input into the CNN. The resultant depth map is optimized by solving a convex energy minimization problem. Unlike Heber and Pock [30] that use only one directional EPI, Shin et al. [12] construct a multi-stream networks for four viewpoints, horizontal, vertical, left and right diagonal directions. They show that their multi-stream network reduces the reconstruction error over single-stream network by 10%. They claim that the use 2 × 2 kernel in the algorithm reduces the effect of noise. In this paper, we compare our results with their multi-stream network on both synthetic images with and without noise, and even though their algorithm out-performs our algorithm for noiseless images, our algorithm out-performs their results for all images with added noise.

## 5. Methodology

**Figure 5:**
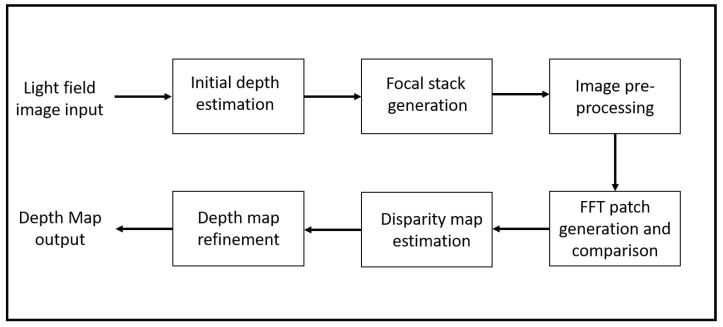
Flow of the proposed algorithm.

The methodology presented in this paper exploits the characteristic of light field images in having multiple focal planes that are captured in a single image. The flow of the algorithm is represented in Figure 5. As shown in Figure 5, the methodology can be divided into four main sections: initial depth estimation, focal stack generation, patch generation and comparison and depth refinement. Unlike the depth from defocus algorithms described in the literature review, our approach uses frequency patch analysis, which makes the algorithm more resilient to noise.

### 5.1. Initial Depth Estimation

The initial depth estimation algorithm only determines the maximum and minimum depth values, rather than the intermediate depth values. The depth or slope difference between two consecutive focal stack images for the initial depth estimation algorithm is taken to be 0.2; experimental evaluation proved that this value sufficiently covers the depth range to permit accurate estimation of periphery depth values. This initial depth estimation stage improves the depth estimation in two ways: firstly, it reduces the computational time as only the relevant focal stack images are generated; secondly, reduces the number of redundant images to then also reduce the possibility of misdetections.

### 5.2. Focal Stack Generation and Image Pre-Processing

**Figure 6:**
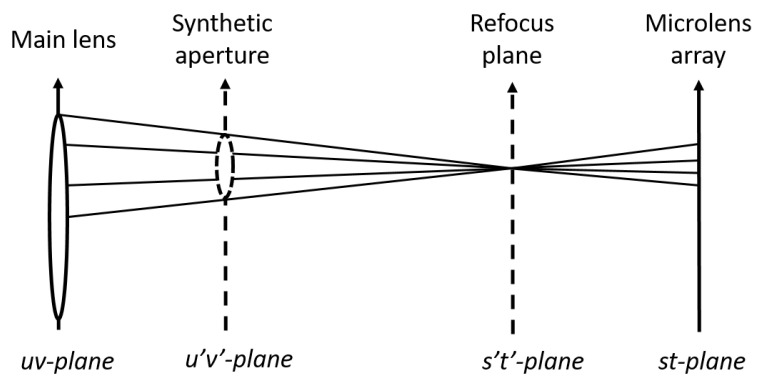
Conceptual model for refocusing LF image at a different depth [17]. The (*u*,*v*) and (*s*,*t*) plane are surfaces of the camera, respectively, and (*s*′,*t*′) is the refocus plane.

A single LF image can be used to generate an all-in-focus image and also to generate the same scene at different focal lengths and narrow depth of field. The sub-aperture images can be obtained by holding (*u*,*v*) fixed and considering all (*s*,*t*) [17]. The central sub-aperture image is the all-in-focus image. Figure 6 shows the conceptual model of a light field image when refocusing at a virtual plane (*s*′,*t*′). Thus, to refocus the image to a different focal plane (*s*′,*t*′), the shifted versions of the sub-aperture images are summed together.

Following the same concept, the focal stack is generated using the shift-sum filter, which is a depth-selective filter that functions like a planar focus. The filter works by shifting (*u*,*v*) slices of the LF image to a common depth and then adding the slices together to obtain the 2D image. The value of the slope controls the amount of shift that corresponds to the image being focused on a different focal plane. The difference in slope values between two consecutive images in the focal stack for our work is 0.01, which implies that to obtain an accurately refocused image at that small depth difference the sub-aperture images have to be shifted at multiples of 0.01 pixels. To shift the sub-aperture images with subpixel accuracy we use a frequency domain approach. In this approach, the relationship between the image shift in the spatial and frequency domains is shown in Equation (1), where *s*_0_ and *t*_0_ is the slope value and *u*, *v* is the sub-aperture location. The amount by which the sup-aperture image has to be shifted to refocus at a particular depth is the product of *s*_0_, *u* and *t*_0_, *v*.

(1) $$\;f\left(s+s_{0},t+t_{0}\right)=F\left(u,v\right)e^{-j2\pi (\frac{us_{0}+vt_{0}}{N})}\;$$

**Figure 7:**
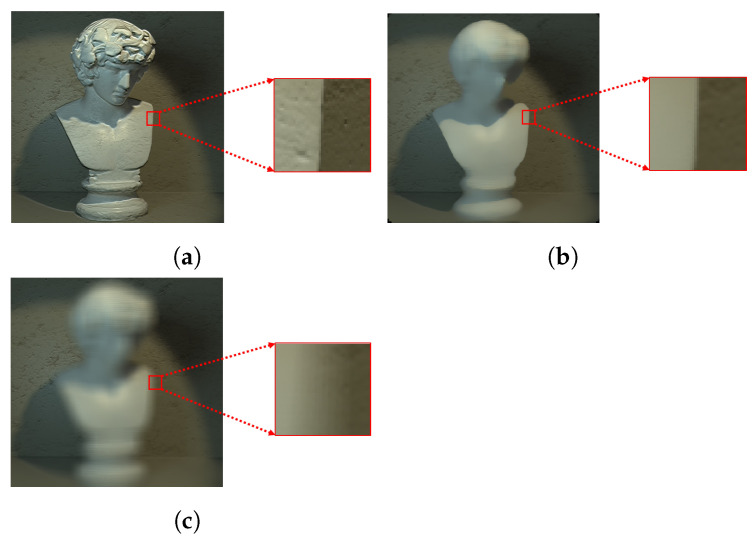
(**a**) Central sub-aperture image of a LF image, (**b**) focal stack image refocused on the background using median pixel values and (**c**) focal stack image refocused on the background using averaged pixel values and magnified region at depth discontinuity for each image.

The refocused images can then be generated by either averaging the shifted sub-aperture pixels or by using the median value of the pixels. Figure 7 shows a comparison of the blur around the depth discontinuities when focusing on the background for both the summing techniques. It is clear from the magnified parts shown in Figure 7a–c that the amount of defocus blur around the depth discontinuities using the averaged pixel value is very large compared to the amount of defocus blur using the median value.

**Figure 8:**
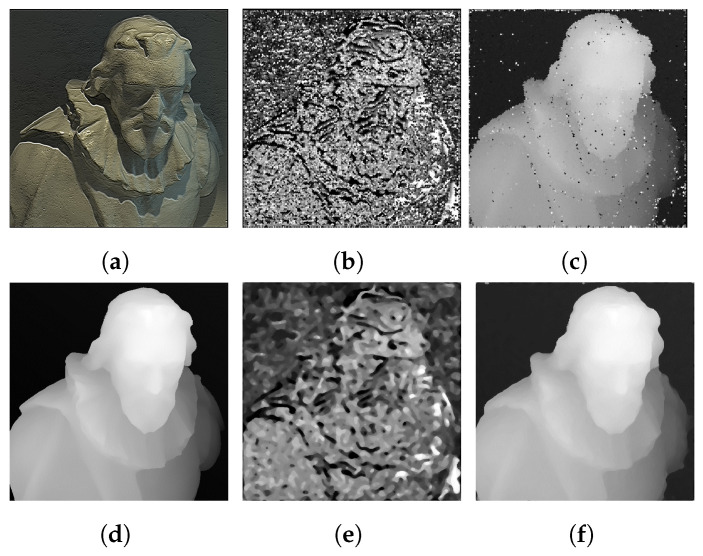
(**a**) Central sub-aperture image of a LF image, (**b**) unrefined depth map calculated without gradient addition, (**c**) unrefined depth map calculated with gradient addition, (**d**) ground-truth depth map, (**e**) refined depth map calculated without gradient addition and (**f**) refined depth map calculated with gradient addition. The Badpix metric for without and with gradient addition is 9.11% and 96.23%, respectively.

The technique used in this paper estimates the depth map by matching image patches from the focal stack to the central sub-aperture image, thus it must be ensured that all the edges and textured regions in the image are well defined in both the central all-in-focus image as well as the focal stack images to minimize the number of misdetections. This problem is addressed by adding the gradient of the image to itself. The main advantage of adding the gradient relies on the fact that in a refocused focal stack images, the textured regions in the image that are in focus maximally contribute to the gradient image, while the out-of-focus objects will contribute the least. This pre-processing step ensures that the object boundaries and textured regions are exaggerated in the focal stack images drastically reducing the number of misdetected patches, in turn reducing the dependence on the post-processing steps. As the purpose of the gradient addition step is only to enhance the textured regions and boundaries on the focal stack images that are in-focus, unless the shadows cause the region to become textureless in the image, our algorithm is not affected by this step. The comparison between the accuracy of the estimated depth map with and without the gradient addition is shown in Figure 8 and it can be seen in Figure 8c,f that the part of the image with shadows are also estimated accurately.

### 5.3. Patch Generation and Comparison

The focal stack images that are acquired in the previous stage are divided into smaller image patches and then those individual patches are compared with the corresponding block in the all-in-focus image. Since the accuracy of the approach is dependent on the similarity check of the individual image patches, it is crucial that a block of appropriate shape and size is selected. Initial tests for patch selection were performed with a square patch of size 10 × 10 pixels. This was the preliminary test that was performed to validate the approach. The test was then repeated with patches of different sizes. The results showed that smaller window sizes covered the image regions and boundaries more accurately. As the window size decreases, the processing time increases as the number of patches that are being compared increase; however, the nature of square-shaped patches over or under-compensated object boundaries that were slanted or curved in the image.

**Figure 9:**
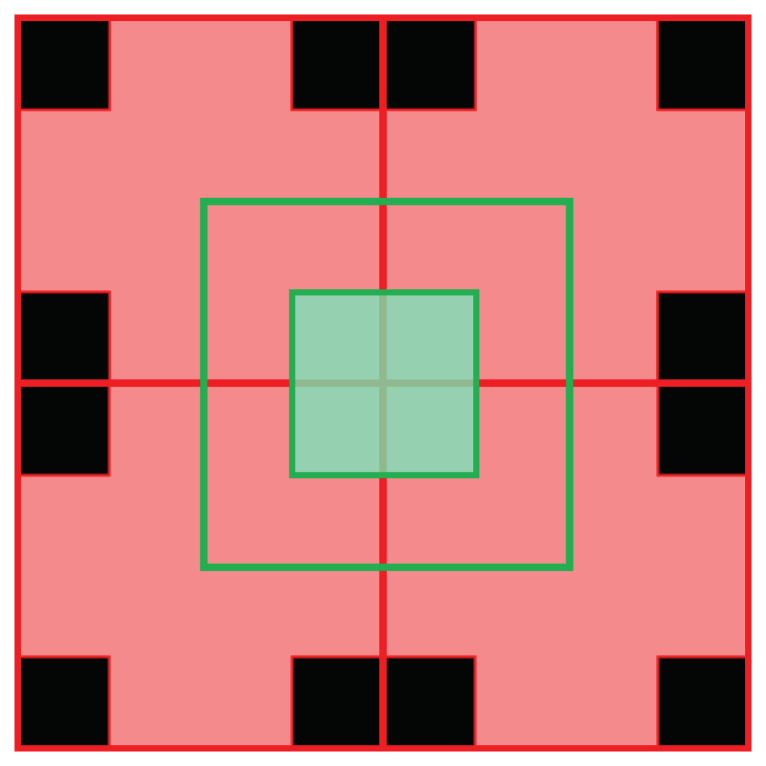
The red and green squares are the two overlapping 4 × 4 pixel patches used to cover the entire image. As the patches overlap, only the highlighted red and the green pixels from the red and green squares are used to estimate the depth.

In testing cross shape patches, the area that is uncovered in the gaps between four consecutive cross is less than that of the primary cross window size. This meant that to cover the entire image without any gaps, cross patches of two different sizes were used. The shape and size of the primary cross governs the shape and size of the secondary cross as shown in Figure 9. To address misdetections due to small window size using cross patches, an overlapping window is used. For the proposed algorithm, we use the two cross patches of size 4 × 4 pixels as shown in Figure 9. In Figure 9 the red and green squares are the pixels that are considered for matching with the all-in-focus image patch, but only the pixels highlighted in red in the red square and the pixels highlighted in green in the green square are used to generate the depth map. The patch size can be reduced lower than 4 × 4 pixels, although experimental tests revealed that using a patch of size smaller than 4 × 4 pixels does not improve the depth map accuracy and increases the computational time.

**Figure 10:**
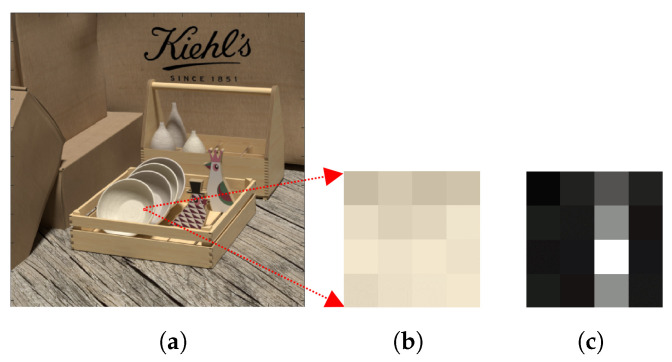
(**a**) Central sub-aperture image of a LF image, (**b**) a magnified 6 × 6 RGB image patch and (**c**) FFT of the image patch.

**Figure 11:**
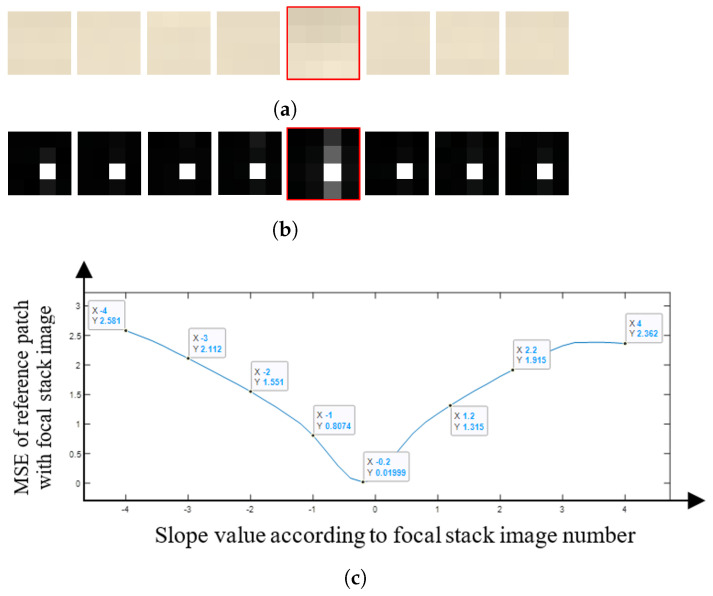
(**a**) The RGB image patch and (**b**) the FFT patch at different focal lengths. The patch with the red boundary is the closest match to the reference patch in Figure 10. (**c**) The graph shows the MSE values for the central image in Figure 10, with the corresponding focal stack image patch.

By comparing the FFT of the image patches, we are no longer looking at individual pixel values when comparing the image patches, but a frequency domain representation of those patches, which makes the comparison more robust to noise. To illustrate the proposed approach Figure 10 shows the central sub-aperture image of the LF image, a 4 × 4 pixel patch taken from the image and the FFT of the patch generated. The focal length for synthetic images lies between a slope of −4 to +4 and for a Lytro camera, the image lies between slopes of −2 to +2. We therefore correspondingly generated the focal stack from a slope of −4 to +4 for synthetic LF images, and from the slope of −2 to +2 for real LF images. The slope interval between two consecutive focal stack images defines the number of depth levels in the depth map. We found through experimentation that for the purposes of our work, the slope can be varied at an interval of 0.01, as using an interval below 0.01 does not show any significant change in the focus for consecutive focal stack images for both the synthetic and real LF images. Thus, for a synthetic LF image the depth map can have up to 801 depth levels as the focal stack is generated from a slope of −4 to +4 at a slope interval of 0.01, while for a real LF image the depth map can have up to 401 depth levels as the focal stack is generated from a slope of −2 to +2 at a slope interval of 0.01. The depth levels for the depth map can be increased by reducing the slope interval between the focal stack images, although as the depth levels increase the computation time also increases. For visual brevity in Figure 11, only 8 refocused images are considered from −4 to +4 slope at an interval of 1. It is clearly seen that the fifth patch in Figure 11 is the most similar to the reference patch.

### 5.4. Depth Map Refinement

**Figure 12:**
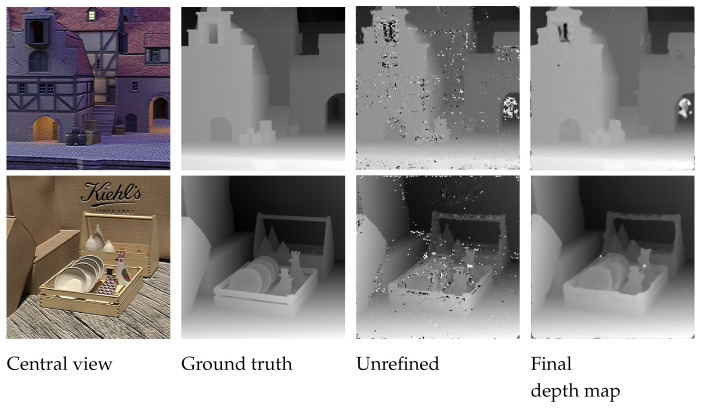
Comparison between the ground truth and the estimated depth map before and after the refinement step for synthetic images.

**Figure 13:**
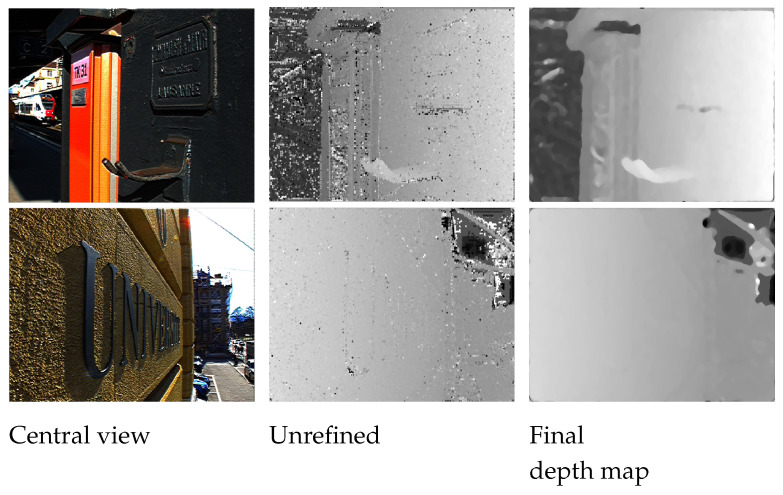
Comparison between the estimated depth map before and after the refinement step for real images.

The depth map evaluated up to this stage has a few patches that are not detected correctly, and since the patches are shaped as a cross, it creates a depth map with jagged edges, thus the object boundaries need to be refined in order to restore the shape of the object. Figure 12 and Figure 13 show the comparison between the ground truth and the estimated depth map before and after this refinement step for the synthetic and real images. The disparity map is refined in two steps using an iterative approach. Firstly, the histogram of the disparity map is checked for the number of pixels that are present at each depth. If the number of pixels at a particular depth falls below the threshold value, those pixels are filled with the maximum likelihood value of the pixels in the depth map at that position using the pixel value that occurs the most times in the neighboring pixels. The second step is similar to the first step, but instead of looking at individual pixels, the cross patches are considered. This step checks for any isolated patches in the image that have different surrounding depth patches. Once these patches are isolated, the patch is filled with the value of pixels with maximum likelihood in the depth map at that patch position using the pixel value that occurs the most times in the neighboring pixels.

## 6. Misdetection Analysis

**Figure 14:**
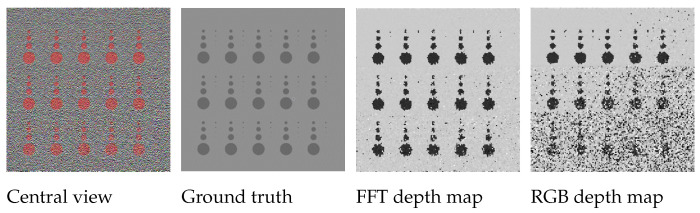
Visual comparison between ground truth depth map, the result using FFT to estimate depth map (proposed algorithm) and depth map estimated using RGB patches.

**Figure 15:**
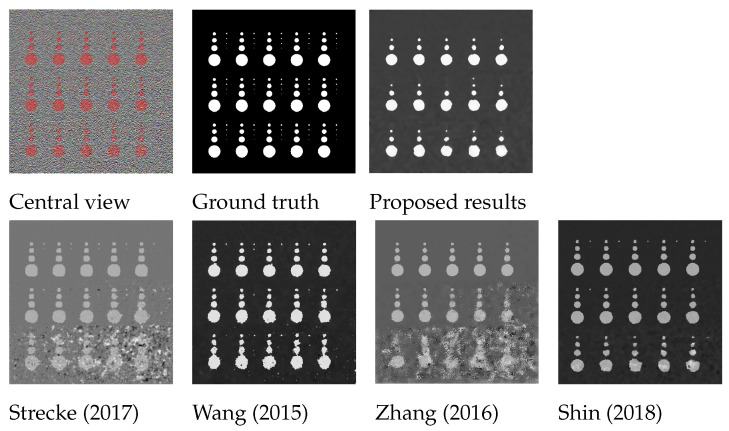
Visual comparison for the dot image of the proposed algorithm with Strecke et al. [9], Wang et al. [10], Zhang et al. [11] and Shin et al. [12] for synthetic LF images.

The depth estimation in the proposed algorithm compares the FFT of the all-in-focus patch and the patches at different focal positions in the focal stack to check for the least error to then accordingly estimate the depth. The quantitative results for ‘dot’ image presented in Table 1 confirm that both techniques give comparable results. The advantage to using the FFT domain over the spatial RGB patch is that the number of misdetections is drastically reduced with the FFT, which reduces the algorithm dependence on the depth map refinement stage. A closer visual comparison in Figure 14 also shows that the depth boundaries are sharper and the depths are more accurately represented for the results using FFT. The ‘dot’ image is one of the more challenging images from the dataset due to the added Gaussian noise to approximate thermal and shot noise [32], and the shape and size of the objects in the image. In further evaluation, image-level comparisons for the proposed algorithm are shown in Figure 14. It can be seen that the noise in the image considerably reduces the depth map accuracy when using RGB patches, where parts of objects in the image are completely misdetected; whereas, for the depth map generated by using the proposed FFT comparison, the results are more noise-resilient. The proposed algorithm also outperforms the state-of-the-art for the ‘dot’ image, as can been seen in Figure 15.

**Figure 16:**
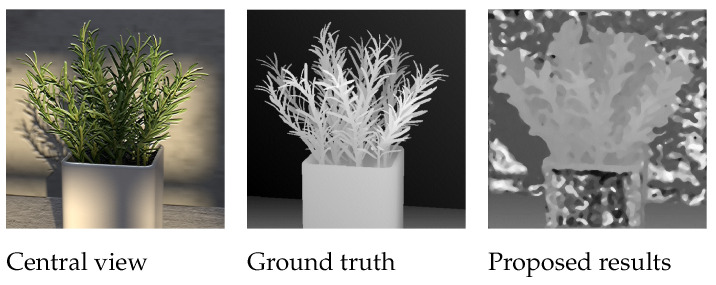
Misdetection of textureless regions in Rosemary image.

Figure 16 shows central view, ground-truth depth map and the estimated depth map for the Rosemary image in the synthetic image dataset. Our algorithm produces an inaccurate depth map for the Rosemary image with a Badpix 0.07 value of 0.34. The error is caused because the wall in the background and the vase in the foreground have a smooth and textureless surface, which make the two indistinguishable by our algorithm. It is important to note that the misdetection is not caused by the shadows in the image as the carpet at the bottom of the image is not misdetected even though shadows falls on the carpet as well, and the cotton image in Figure 8 also show that shadows do not effect the depth map accuracy for our algorithm.

**Figure 17:**
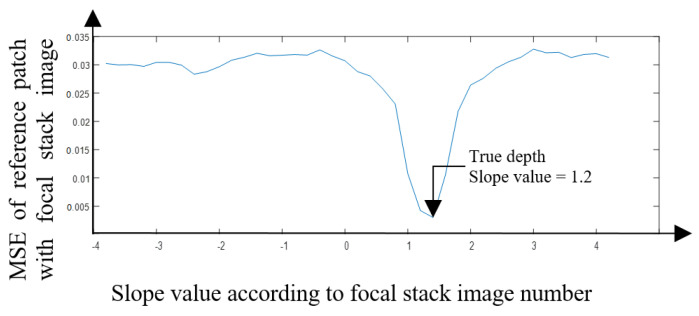
MSE for the central image patch and the patch at different focal lengths when the depth is estimated correctly.

**Figure 18:**
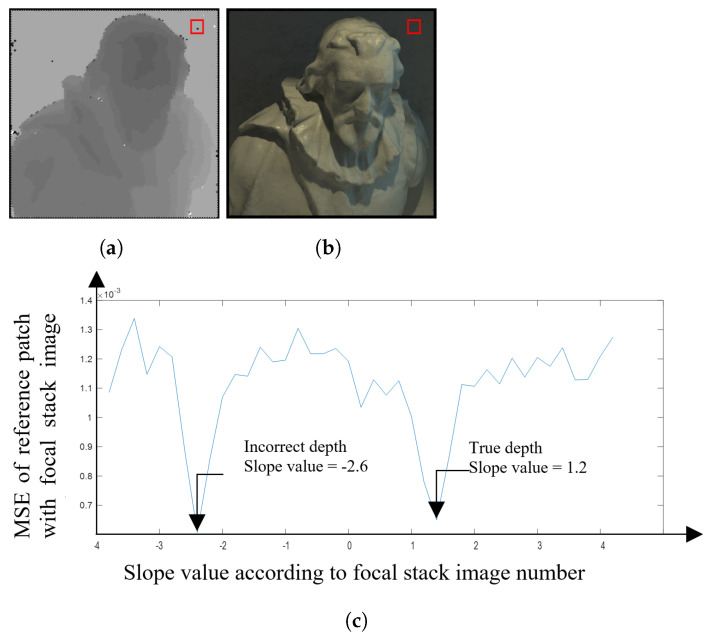
(**a**) The depth map of cotton image with the red square showing the cross error patch. (**b**) The central image with the red box showing the error patch. (**c**) The MSE of the central image patch with the patch at different focal length.

Figure 17 shows the mean squared error (MSE) for the patch that is estimated to the correct depth at different focal lengths with the patch of the all-in-focus image. This trend in the graph indicates that the image patch is depicting the correct depth: in the example shown in Figure 17, the plot takes a significant dip as it reaches the least MSE, which is the true depth for that particular patch. As the patch goes further away from the correct depth value, the graph makes a similar curve on both the sides of the focal stack. Even though in our work we are only using the patches with the least MSE to estimate the depth map, it is an important observation to see that the graph almost traces a bell curve. This shows that the MSE value is similar when defocusing toward or away from the patch in focus. In contrast, Figure 18 shows an example of a patch being misdetected in the ‘cotton’ image. Although the graph follows a similar trend to Figure 17, the graph has two considerable dips, one at the correct depth of slope 1.2, and the other at the incorrect depth of slope −1.6. It is also important to note that the number of misdetections are less even though the depth map has not been refined at this stage.

## 7. Experimental Results

**Figure 19:**
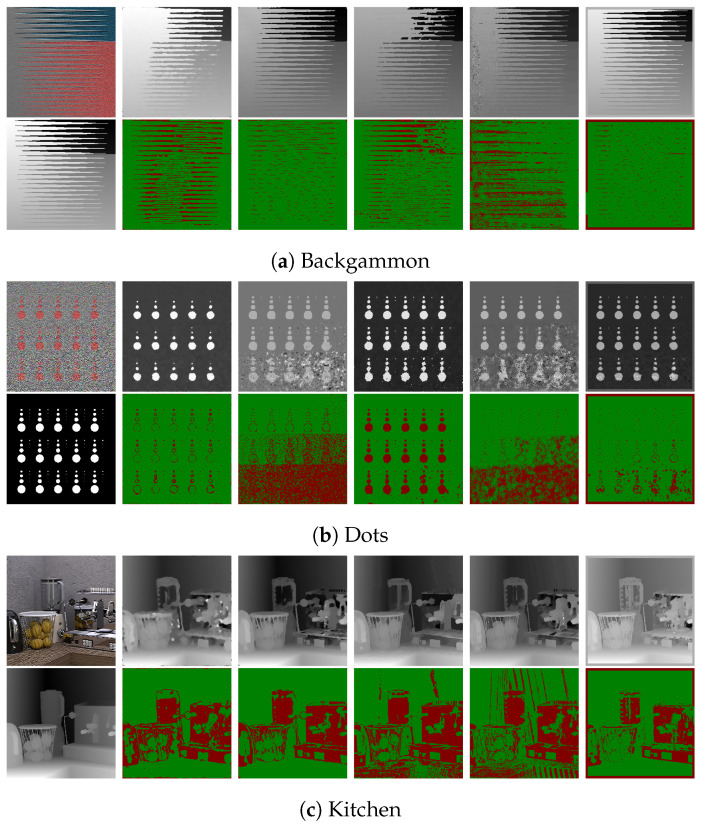

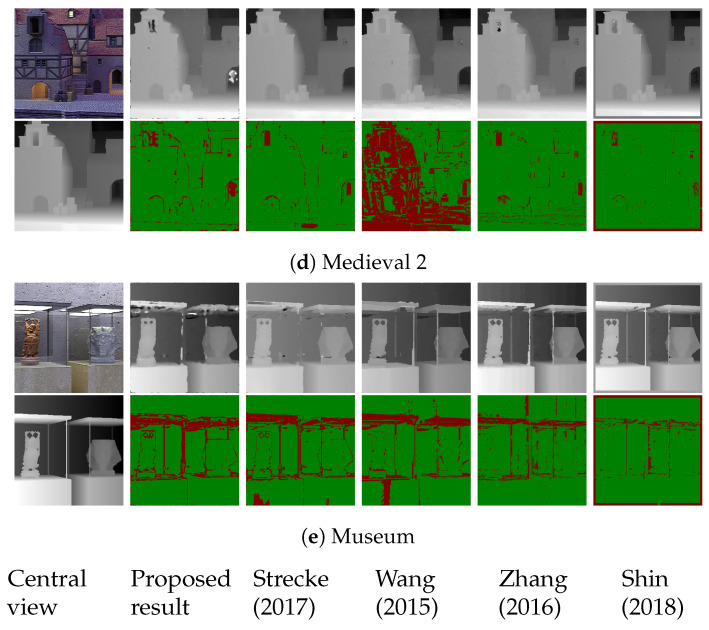
Visual comparison of the proposed algorithm with Strecke et al. [9], Wang et al. [10], Zhang et al. [11] and Shin et al. [12] for synthetic LF images.

**Figure 20:**
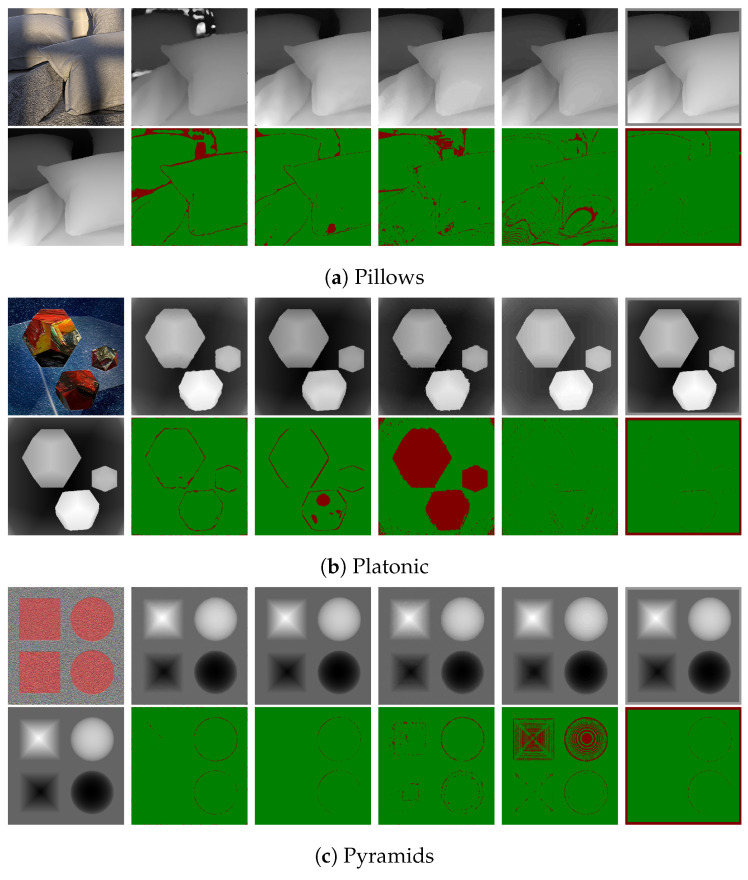

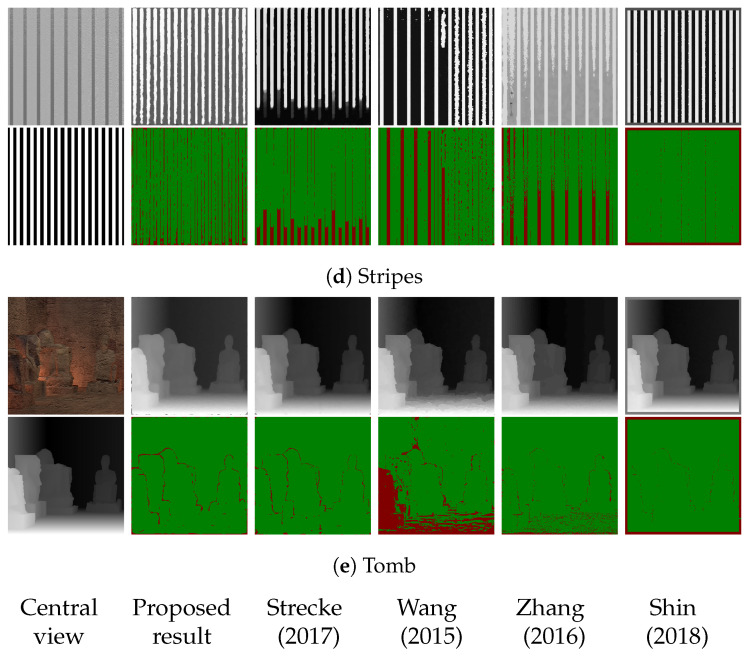
Visual comparison of the proposed algorithm with Strecke et al. [9], Wang et al. [10], Zhang et al. [11] and Shin et al. [12] for synthetic LF images.

**Figure 21:**
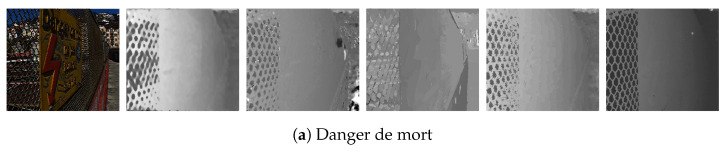

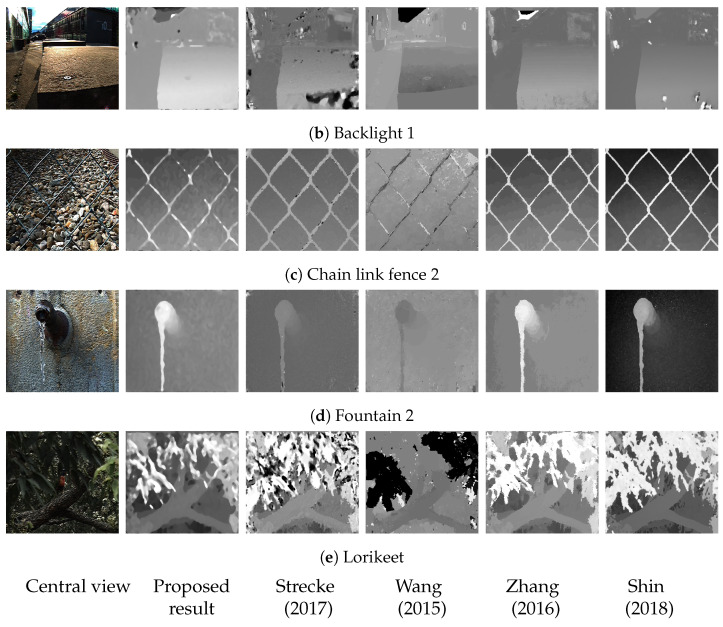
Visual comparison of the proposed algorithm with Strecke et al. [9], Wang et al. [10], Zhang et al. [11] and Shin et al. [12] for real LF images.

**Figure 22:**
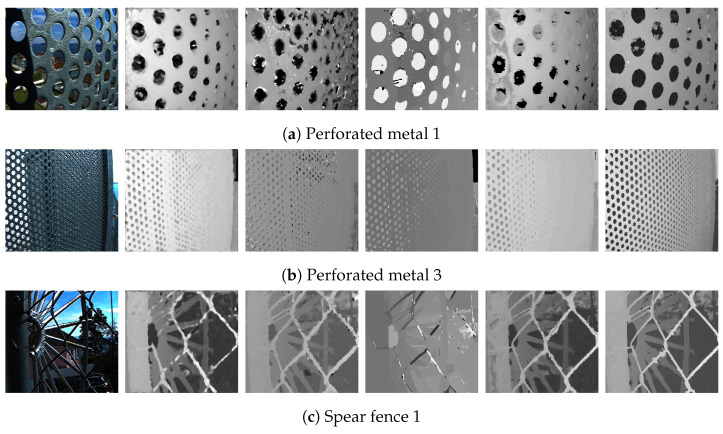

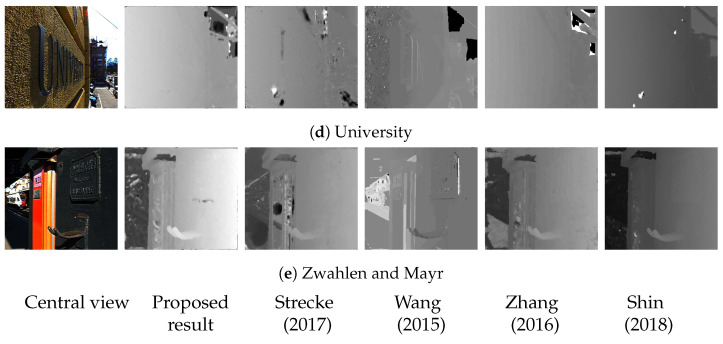
Visual comparison of the proposed algorithm with Strecke et al. [9], Wang et al. [10], Zhang et al. [11] and Shin et al. [12] for real LF images.

The results of the proposed algorithm were evaluated on both real and synthetic light field image datasets. For the synthetic data, the 4D light field dataset [32] was used. The dataset is widely used for validations of depth estimation algorithms for light field images as it contains ground-truth disparity and depth maps. The dataset contains 28 image with a 9 × 9 sub-aperture images with a resolution of 512 × 512. For evaluation of our algorithms with the benchmark algorithms we have selected 10 images as each image contains different materials, lighting conditions and complex structures. Figure 19a–c and Figure 20d have finer detail and complex occlusions. Figure 19c,d have transparent and reflective surfaces. Figure 19d and Figure 20a,e have shadows. Figure 19b and Figure 20b,c are abstract scenes with complex textures. For the real data the EPFL light field dataset [33] was used. The real image dataset contains 138 LF images in LFR (light field raw) file format captured by a Lytro Illum camera with a 15 × 15 sub-aperture images with a resolution of 434 × 625. The Lytro Illum camera used for LF image acquisition have different calibration data and the LFR files were processed by their corresponding calibration data. Figure 21a,c and Figure 22a–c contain finer objects and complex surfaces such as perforated metal and fences. Figure 21b and Figure 22c,d contain textureless or overexposed regions such as the sky. Figure 21a,b and Figure 22d,e show a gradual change in depth and Figure 21e contains complex structures such as the branches and trees.

The depth maps generated by our proposed approach initially calculates the depth range, and the depth levels vary according to the maximum and minimum depth values present in each LF image. The slope for real data is within the range of +2 to −1, whereas for the synthetic data lies within the range of −1 to +4 and the slope interval used for both type of images is 0.01, as explained in Section 5.2 and Section 5.3. The number of depth levels can be increased or decreased by reducing or increasing the slope interval between focal stack images. The proposed algorithm is compared to four benchmark techniques from Strecke et al. [9], Wang et al. [10], Zhang et al. [11] and Shin et al. [12] using the BadPix metric that specifies the percentage of pixels where disparity deviates by less than 0.07, 0.03 and 0.01 pixels from the ground truth. We have chosen these four techniques are they are the state-of-the-art for the different depth estimation techniques. Strecke et al. [9] and Wang et al. [10] use depth from defocus, Zhang et al. [11] use EPIs, whereas Shin et al. [12] use CNN for depth estimation. In order to compare the depth maps using different algorithms to the ground truth, all output disparity maps are normalized to the ground truth depth map range. For Strecke et al. [9] and Shin et al. [12], normalized results are directly compared to the ground-truth disparity map. For Wang et al. [10] and Zhang et al. [11] the disparity map is normalized before comparing it to the ground truth.

### 7.1. Synthetic LF Images

The LF images in the 4D Light Field Dataset [32] comprise 9 × 9 sub-aperture images. For the synthetic images, the images in Figure 19 and Figure 20 show the error pixels in red where depth deviates by more than 0.07 from the ground truth and the pixels in green where depth deviates by less than 0.07. Table 2 and Table 3 compares the ground truth images to the disparity maps generated by the algorithms that are being tested using the Badpix metric. The LF images generated synthetically have little to no noise compared to the real LF images, and thus the estimated depth maps have less misdetections and the depth boundaries are well defined on the synthetic data compared to the real data. Table 2 and Table 3, which are a comparison of the depth maps with the ground-truth depth map, show that the proposed algorithm out-performs the state-of-the-art algorithms in the two criteria of Badpix 0.07 and 0.03 for the ‘dots’ images from the synthetic images in the dataset. On visual inspection of Figure 19 and Figure 20, it shows that even though the noise level is increased in the bottom part of the image, the background is region is still detected accurately. For ‘medieval 2’ image, the region in the image near the window on the top left and near the door on the bottom right has a dark shadow, which is the common area misdetected for all algorithms. Shin et al. [12]’s produce the least errors around object boundary for the synthetic light field images. The ‘kitchen’ and ‘museum’ image in Figure 19c,e shows how the error pixels are in the same regions for all the estimated depth maps. The reason for the similarity is those regions in the image are either transparent or reflective surfaces. Shin et al. [12] show fewer errors around these regions as they explicitly use a mask for these type of surfaces while training their network. The depth map for our algorithm, Shin et al. [12] and Strecke et al. [9] give similar results for the background and foreground region in the ‘kitchen’ and ‘museum’ image, whereas the depth maps from Wang et al. [10] and Zhang et al. [11] produce errors in the background and foreground regions. For the ‘museum’ image in Figure 19e, and the ‘pillow’, ‘platonic’ images in Figure 20a,b, our proposed algorithm out-performs the non-CNN based algorithms at Badpix 0.03, with comparable results for the other two criteria. Out of the three images mentioned above, for the ‘museum’ image the main reason for the errors is the reflective display case and the bright display case lighting. For the ‘platonic’, ‘pyramids’ and ‘tomb’ images in Figure 20, our depth map generates error only at depth boundaries and all other regions are estimated accurately and is comparable to Shin et al. [12]’s CNN approach. Shin et al. [12] produce high accuracy depth maps and are also able to distinguish accurate depths for occlusions and smaller objects in the image, but the accuracy reduces for transparent or reflective surfaces and for noisy images.

### 7.2. Real LF Images

The proposed algorithm is not able to distinguish objects in the image that are less than 4 × 4 pixels in width due to the patch size used, but using patches of size less than 4 × 4 pixels drastically increases the number of misdetected depth patches and also increases the computational time. The image result displayed in Figure 21 and Figure 22 on visual inspection shows similar outcomes as for the synthetic images. The central view for four images in the Figure 21a,b and Figure 22d,e show a gradual change in depth and the depth maps correspondingly show the gradient change. For the proposed algorithm, the images in Figure 21a,c and Figure 22c with the chain fences, the regions where the chain has a shadow cast over it is misdetected. The chain fences in all three images for all the algorithms have been under or over-compensated. The lorikeet image in Figure 22e is a complex image with the leaves and branches, but the proposed algorithm performs comparatively with Strecke et al. [9] and Zhang et al. [11]; on the other hand, with Wang et al. [10] major parts of the image are misdetected. For the ‘perforated metal 1’ image Figure 22a, parts of the image in the far background and foreground are represented with less error as compared to all the other depth maps, whereas in the ‘perforated metal 3’ image Figure 22b, Wang et al. [10] better estimates the depth around the holes in the metal frame. In Figure 21 and Figure 22, misdetections can been seen for textureless regions for the ‘backlight 1’ image Figure 21b, the ‘perforated metal’ image Figure 22a, and the ‘university’ image Figure 22d, i.e., the regions of the image with the sky.

### 7.3. Noisy Image Analysis

**Figure 23:**
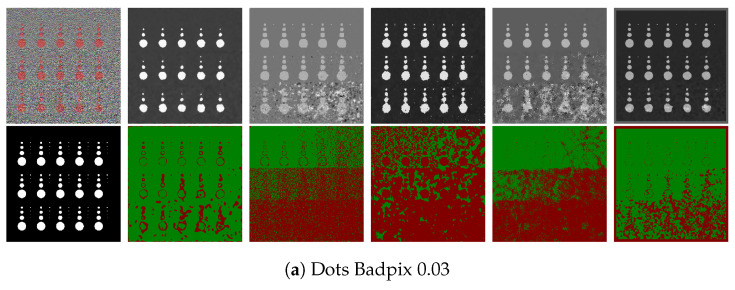

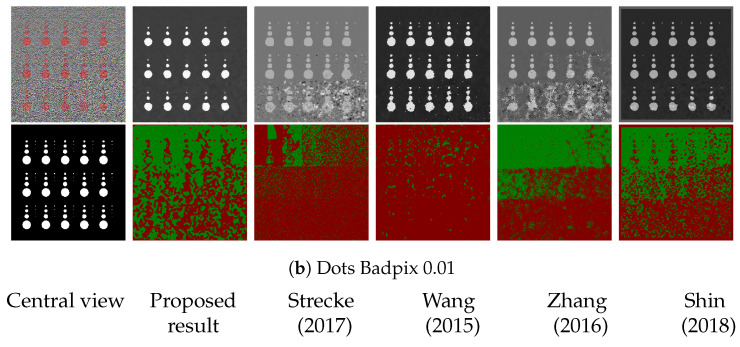
Visual comparison of the ground truth with the proposed algorithm, Strecke et al. [9], Wang et al. [10], Zhang et al. [11] and Shin et al. [12] for dots image for Badpix 0.03 and 0.01.

The results in Figure 15 and Figure 23 and Table 4 demonstrate that the proposed algorithm is more noise-resilient than existing approaches. To further explore our algorithm’s noise resilience, Gaussian noise approximates thermal and shot noise in images, and Gaussian noise with zero mean and variance of 0.01 was added to the 4D Light Field Dataset [32]. Gaussian noise was chosen as it approximates thermal and shot noise in images, as at large light levels, the Poisson distribution that describes shot noise approaches a normal distribution and can be approximated using Gaussian noise.

**Figure 24:**
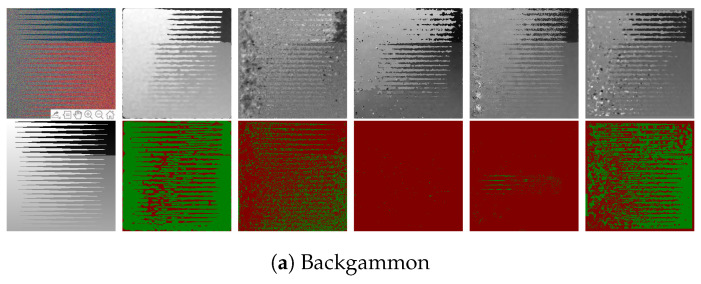

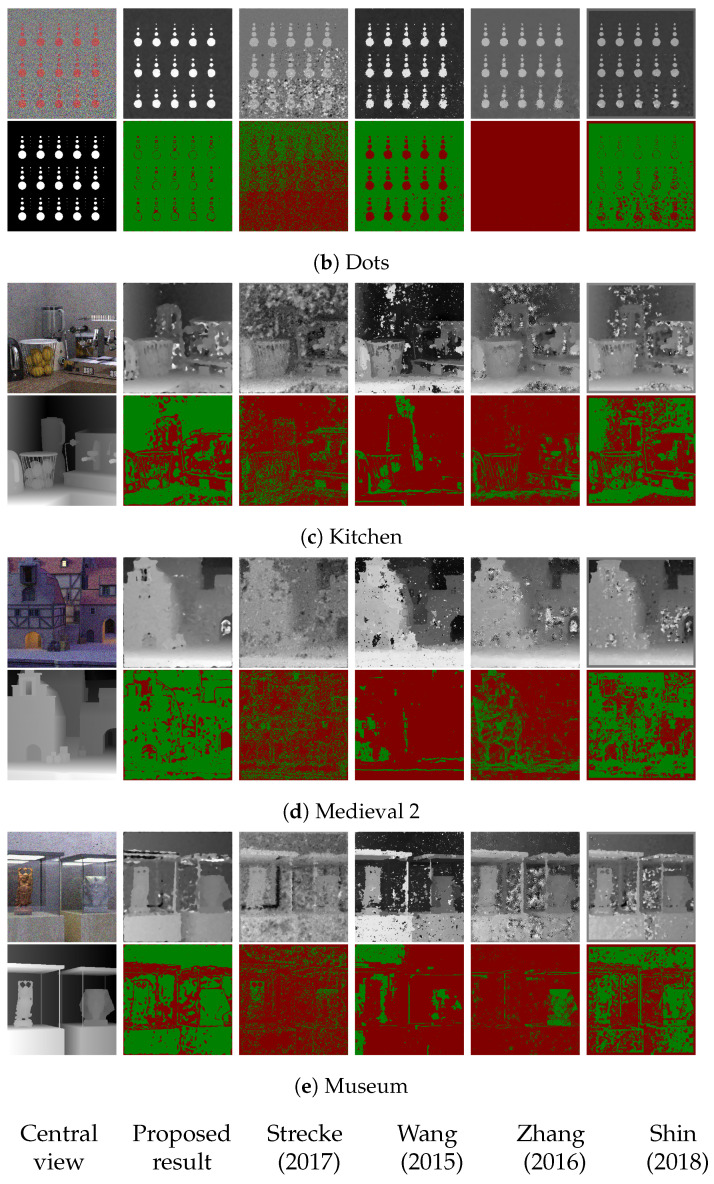
Visual comparison of the proposed algorithm with Strecke et al. [9], Wang et al. [10], Zhang et al. [11] and Shin et al. [12] for synthetic LF images with added noise.

**Figure 25:**
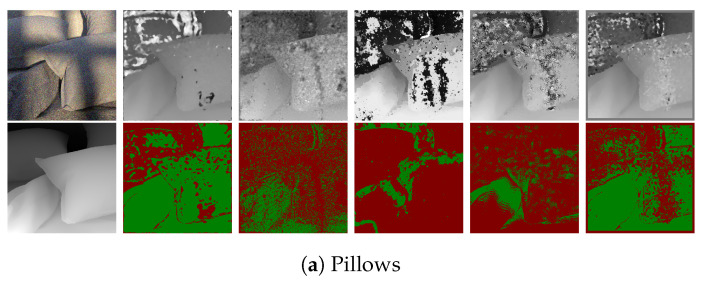

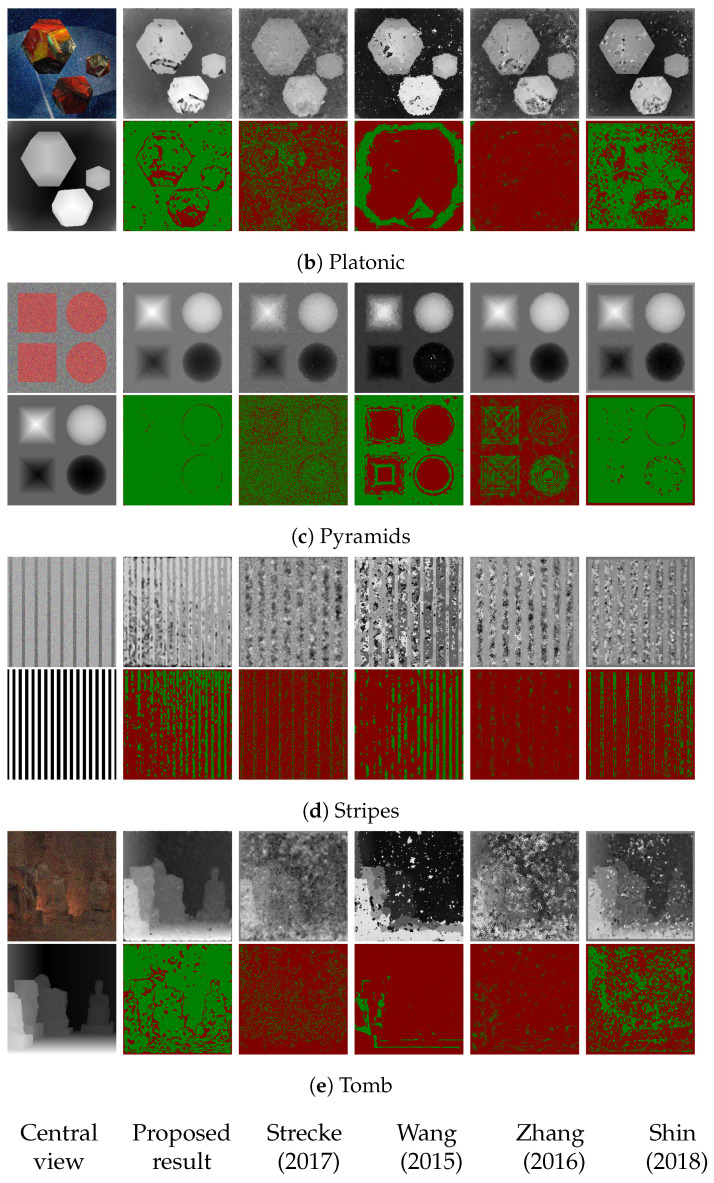
Visual comparison of the proposed algorithm with Strecke et al. [9], Wang et al. [10], Zhang et al. [11] and Shin et al. [12] for synthetic LF images with added noise.

The proposed algorithm out-performs the benchmark algorithms for all images in all three criteria as shown in Table 5 and Table 6, Figure 24 and Figure 25. The algorithm from Zhang et al. [11] and Wang et al. [10] generates a disparity map that uses the maximum and minimum depth values from the ground-truth depth map to scale the disparity map accordingly. The problem with noisy images is that misdetections and outliers make the re-scaling unreliable and nontrivial. It is clear from the results shown in Table 5 and Table 6 that the accuracy of the proposed algorithm is also significantly affected for most of the images, but the algorithm is still able to estimate a depth map with comparatively high accuracy compared to the state-of-the-art algorithms. The average accuracy for our algorithm for Badpix 0.07 across the ten images used for testing shown in Figure 24 and Figure 25 is 0.75, whereas for Shin et al. [12], Strecke et al. [9], Wang et al. [10] and Zhang et al. [11] it is 0.6, 0.3, 0.26 and 0.11, respectively. For the ‘dots’, and ‘pyramids’ images the accuracy is over 95% out-performing the state-of-the-art algorithms. The images with finer details such as the ‘backgammon’ image in Figure 24a has a considerable amount of misdetections, but algorithm is still able to obtain a Badpix 0.07 value of 0.74. The ‘kitchen’ and ‘museum’ image in Figure 24c,e shows errors for transparent or reflective surfaces, but the depth map for our algorithm estimates the background and foreground region in the image accurately with Badpix 0.07 value of 0.63 and 0.69, whereas the depth map from Wang et al. [10] and Zhang et al. [11] produces errors in the background and foreground regions and Badpix 0.07 value below 0.25. Shin et al. [12], on the other hand, shows an Badpix 0.07 value 0.5 and 0.52, but large parts of the background and foreground region are misdetected. An important observation that can be drawn from Table 2, Table 3, Table 5 and Table 6 is that without the added noise the average accuracy for the proposed algorithm, Shin et al. [12], Strecke et al. [9], Wang et al. [10] and Zhang et al. [11] is 0.96, 0.9, 0.89, 0.79 and 0.86, respectively. After the noise is added to the images, our accuracy reduces to 0.75, whereas for Shin et al. [12] it reduces to 0.6, Strecke et al. [9] it reduces to 0.3, Wang et al. [10] it reduces to 0.26 and Zhang et al. [11] it reduces to 0.11.

### 7.4. Runtime Complexity Analysis

The code for the proposed algorithm was implemented in MATLAB^TM^ on an Intel i7 machine at 1.9 GHz and 16 GB of RAM. The results for Strecke et al. [9], Wang et al. [10] and Zhang et al. [11] were generated using the same machine. Table 7 and Table 8 show the average runtime for synthetic images and real images, respectively, over all the images in the dataset for a single run for the proposed algorithm compared to the state-of-the-art algorithms. The runtime for Strecke et al. [9] for real images is lesser than for synthetic images, as running their complete code requires the parameter files that are not available for the real images used. As Shin et al. [12] propose a CNN approach, their network has to be trained and their network takes 5 days to train.

The runtime for the proposed algorithm varies for each of the images as the algorithm first calculates the maximum and minimum depths for the image and then generates the focal stack. The runtime for the algorithm can be divided into four stages: the first stage calculates the initial depth, which takes an average of 52.18 s; all of the pre-processing steps in the second stage for the images takes an average of 0.21 s per image; the third stage generates the focal stack where majority of the processing time is spent, an average duration of 2.17 s per image; and the last stage is where the depth map is estimated and refined, which on average is 0.8 s per image. For real images the initial depth estimation stage takes an average of 150 s; all the pre-processing steps for the images takes an average of 0.21 s per image; the third stage generates the focal stack where majority of the processing time is spent, an average duration of 9.68 s image, and the last stage estimating and refining the depth map on average takes 1.02 s per image. The run time can be approximated by adding the per image times for each stage and multiplying it by the number of focal stack images. The runtime for the real images is more than the synthetic images as the number of sub-aperture images is more in the case of real images.

## 8. Conclusions

Depth maps from light field images are essential for light field image compression techniques, reconstructing views from a sparse set of perspective views, increasing the number of perspective views and 3D reconstruction. In this paper, we proposed a depth estimation algorithm that works on the concept of depth from defocus. Our experimental evaluation shows that the proposed algorithm outperforms state-of-the-art algorithms for both synthetic and the real light field image datasets. We also show that comparing the FFT of the images patches instead of the RGB patch directly increases our algorithm’s resilience to noise.

One key advantage of our algorithm is that it can be used to estimate the depth map using focal stack images captured by a 2D camera. The limitation of the proposed methodology is that the size of the patches makes it difficult to distinguish objects smaller than 4 × 4 pixels. To address this limitation we are currently investigating how the algorithm would perform if we would increase the resolution of the LF image or the focal stack images.

## Figures and Tables

**Table 1:** Comparison of the estimated depth map when using the FFT Patch and RGB Patch algorithms.

	Dots	Dots
	FFT Patch Depth Map	RGB Patch Depth Map
Badpix 0.07	**0.9705**	0.7760
Badpix 0.03	**0.8853**	0.5967
Badpix 0.01	**0.3880**	0.2391

**Table 2:** Quantitative depth map comparison for synthetic data to ground truth for different algorithms.

	Back-Gammon	Dots	Kitchen	Medi-eval2	Museum
Proposed Results					
Badpix7	0.8230	**0.9605**	0.7010	0.9362	0.8440
Badpix3	0.7324	**0.8853**	0.5941	0.8528	0.7772
Badpix1	0.4910	0.3880	0.3749	0.5514	0.5305
Strecke et al. [9]					
Badpix7	0.9580	0.6273	0.7224	0.9608	0.8578
Badpix3	0.9283	0.4514	0.6282	0.8895	0.7615
Badpix1	0.6606	0.1777	0.4644	0.6469	0.5256
Wang et al. [10]					
Badpix7	0.8753	0.8801	0.6300	0.5136	0.8522
Badpix3	0.4525	0.2485	0.3991	0.1119	0.6902
Badpix1	0.0544	0.0456	0.1772	0.0370	0.2741
Zhang et al. [11]					
Badpix7	0.7889	0.7358	0.6379	0.9580	0.8940
Badpix3	0.3762	0.4810	0.3165	0.7513	0.5413
Badpix1	0.1057	0.4810	0.0997	0.2658	0.1899
Shin et al. [12]					
Badpix7	0.9777	0.9473	0.7931	0.9847	0.9598
Badpix3	0.9594	0.7957	0.7209	0.9584	0.9053
Badpix1	0.8265	0.5122	0.4809	0.7263	0.6478

**Table 3:** Quantitative depth map comparison for synthetic data to ground truth for different algorithms.

	Pillows	Platonic	Pyramids	Stripes	Tomb
Proposed Results					
Badpix7	0.9212	0.9747	0.9920	0.8853	0.9696
Badpix3	0.8769	0.9447	0.9582	0.8275	0.9100
Badpix1	0.6096	**0.7600**	0.7485	0.6732	0.6423
Strecke et al. [9]					
Badpix7	0.9710	0.9645	0.9969	0.8741	0.9813
Badpix3	0.8687	0.9230	0.9927	0.8556	0.9252
Badpix1	0.4914	0.7792	0.9417	0.4925	0.6875
Wang et al. [10]					
Badpix7	0.9387	0.6583	0.9843	0.8231	0.7953
Badpix3	0.5611	0.4620	0.7520	0.0048	0.4134
Badpix1	0.1492	0.1889	0.0737	0.0004	0.1359
Zhang et al. [11]					
Badpix7	0.9398	0.9906	0.8958	0.8373	0.9622
Badpix3	0.5066	0.7454	0.1885	0.5243	0.7500
Badpix1	0.1869	0.2946	0.0634	0.5243	0.2871
Shin et al. [12]					
Badpix7	0.9939	0.9981	0.9972	0.9894	0.9963
Badpix3	0.9772	0.9941	0.9917	0.9865	0.9826
Badpix1	0.7727	0.7273	0.8673	0.8869	0.6453

**Table 4:** Comparison for the ‘dot’ image with ground truth for proposed and state-of-the-art algorithms.

Dots					
	**Proposed Result**	**Strecke et al. [9]**	**Wang et al. [10]**	**Zhang et al. [11]**	**Shin et al. [12]**
Badpix7	**0.9605**	0.6273	0.8800	0.7357	0.9473
Badpix3	**0.8853**	0.4514	0.2485	0.4810	0.7957
Badpix1	0.3880	0.1777	0.0456	0.4810	0.5122

**Table 5:** Quantitative depth map comparison for synthetic data to ground truth for different algorithms for noisy images.

	Back-Gammon	Dots	Kitchen	Medi-eval2	Museum
Proposed Results					
Badpix7	**0.7408**	**0.9620**	**0.6341**	**0.8171**	**0.6921**
Badpix3	**0.5126**	**0.8561**	**0.4323**	**0.5584**	**0.4889**
Badpix1	**0.2101**	0.2808	**0.1733**	**0.2290**	**0.2023**
Strecke et al. [9]					
Badpix7	0.2781	0.3975	0.2309	0.3024	0.1938
Badpix3	0.1312	0.1895	0.1140	0.1419	0.0919
Badpix1	0.0450	0.0634	0.0402	0.0487	0.0318
Wang et al. [10]					
Badpix7	0.0022	0.8619	0.1229	0.0892	0.1868
Badpix3	0.0008	0.7684	0.0570	0.0340	0.0790
Badpix1	0.0003	0.1351	0.0197	0.0083	0.0260
Zhang et al. [11]					
Badpix7	0.0144	0.0002	0.1587	0.2456	0.1054
Badpix3	0.0057	0.0001	0.0666	0.1022	0.0517
Badpix1	0.0019	0.0000	0.0217	0.0322	0.0174
Shin et al. [12]					
Badpix7	0.5778	0.8990	0.5035	0.6512	0.5237
Badpix3	0.3265	0.6624	0.3090	0.3898	0.3112
Badpix1	0.1162	0.3034	0.1247	0.1451	0.1181

**Table 6:** Quantitative depth map comparison for synthetic data to ground truth for different algorithms for noisy images.

	Pillows	Platonic	Pyramids	Stripes	Tomb
Proposed Results					
Badpix7	**0.6823**	**0.8457**	**0.9891**	**0.3582**	**0.8008**
Badpix3	**0.5099**	**0.6272**	**0.9108**	**0.1982**	**0.5089**
Badpix1	**0.2417**	**0.2600**	**0.4835**	**0.0930**	**0.1877**
Strecke et al. [9]					
Badpix7	0.3212	0.3093	0.6557	0.1388	0.1404
Badpix3	0.1698	0.1476	0.3456	0.0641	0.0615
Badpix1	0.0613	0.0511	0.1136	0.0212	0.0206
Wang et al. [10]					
Badpix7	0.1472	0.2515	0.6136	0.2488	0.0561
Badpix3	0.0700	0.1126	0.0870	0.1106	0.0209
Badpix1	0.0257	0.0366	0.0141	0.0014	0.0069
Zhang et al. [11]					
Badpix7	0.1794	0.0394	0.2569	0.0419	0.0530
Badpix3	0.0814	0.0148	0.0917	0.0219	0.0214
Badpix1	0.0273	0.0045	0.0115	0.0078	0.0075
Shin et al. [12]					
Badpix7	0.6000	0.6621	0.9729	0.2084	0.3957
Badpix3	0.4383	0.3836	0.8325	0.1051	0.1904
Badpix1	0.2193	0.1461	0.4132	0.0372	0.0664

**Table 7:** Average runtime for synthetic data.

Synthetic Light Field Dataset				
**Proposed**	**Strecke et al. [9]**	**Wang et al. [10]**	**Zhang et al. [11]**	**Shin et al. [12]**
1418 s	462 s	243 s	537 s	7 s

**Table 8:** Average runtime for real data.

Real Light Field Dataset				
**Proposed**	**Strecke et al. [9]**	**Wang et al. [10]**	**Zhang et al. [11]**	**Shin et al. [12]**
2559 s	187 s	658 s	1413 s	8.43 s

## Data Availability

The results presented in the paper are available at https://github.com/rishabhsharma27/Depth_estimation_results, accessed on 23 December 2021.
